# Examining amyloid reduction as a surrogate endpoint through latent class analysis using clinical trial data for dominantly inherited Alzheimer's disease

**DOI:** 10.1002/alz.13735

**Published:** 2024-02-23

**Authors:** Guoqiao Wang, Yan Li, Chengjie Xiong, Tammie L. S. Benzinger, Brian A. Gordon, Jason Hassenstab, Andrew J. Aschenbrenner, Eric McDade, David B. Clifford, Jorge J. Libre‐Guerra, Xinyu Shi, Catherine J. Mummery, Christopher H. van Dyck, James J. Lah, Lawrence S. Honig, Gregg Day, John M. Ringman, William S. Brooks, Nick C. Fox, Kazushi Suzuki, Johannes Levin, Mathias Jucker, Paul Delmar, Tobias Bittner, Randall J. Bateman, for the DIAN‐TU Study Team

**Affiliations:** ^1^ Washington University, School of Medicine St. Louis Missouri USA; ^2^ Dementia Research Centre University College London London UK; ^3^ Yale University School of Medicine New Haven Connecticut USA; ^4^ Emory University Medical Center Atlanta Georgia USA; ^5^ Columbia University Irving Medical Center New York New York USA; ^6^ Mayo Clinic Jacksonville Jacksonville Florida USA; ^7^ Department of Neurology Keck School of Medicine of USC Los Angeles California USA; ^8^ Neuroscience Research Australia, Randwick NSW Australia, and School of Clinical Medicine University of New South Wales Randwick New South Wales Australia; ^9^ National Defense Medical College Saitama Japan; ^10^ Department of Neurology Ludwig‐Maximilians‐Universität München Munich Germany; ^11^ German Center for Neurodegenerative Diseases Munich Germany; ^12^ Munich Cluster for Systems Neurology (SyNergy) Munich Germany; ^13^ Department of Cellular Neurology Hertie Institute for Clinical Brain Research University of Tübingen Tübingen Germany; ^14^ German Center for Neurodegenerative Diseases (DZNE) Tübingen Germany; ^15^ F.Hoffmann‐LaRoche, Ltd. Basel Switzerland; ^16^ Genentech, Inc., a member of the Roche Group South San Francisco California USA

**Keywords:** autosomal dominant Alzheimer's disease, Dominantly Inherited Alzheimer Network, gantenerumab, latent class analysis, solanezumab, surrogate biomarker

## Abstract

**INTRODUCTION:**

Increasing evidence suggests that amyloid reduction could serve as a plausible surrogate endpoint for clinical and cognitive efficacy. The double‐blind phase 3 DIAN‐TU‐001 trial tested clinical and cognitive declines with increasing doses of solanezumab or gantenerumab.

**METHODS:**

We used latent class (LC) analysis on data from the Dominantly Inherited Alzheimer Network Trials Unit 001 trial to test amyloid positron emission tomography (PET) reduction as a potential surrogate biomarker.

**RESULTS:**

LC analysis categorized participants into three classes: amyloid no change, amyloid reduction, and amyloid growth, based on longitudinal amyloid Pittsburgh compound B PET standardized uptake value ratio data. The amyloid‐no‐change class was at an earlier disease stage for amyloid amounts and dementia. Despite similar baseline characteristics, the amyloid‐reduction class exhibited reductions in the annual decline rates compared to the amyloid‐growth class across multiple biomarker, clinical, and cognitive outcomes.

**DISCUSSION:**

LC analysis indicates that amyloid reduction is associated with improved clinical outcomes and supports its use as a surrogate biomarker in clinical trials.

**Highlights:**

- We used latent class (LC) analysis to test amyloid reduction as a surrogate biomarker.
- Despite similar baseline characteristics, the amyloid‐reduction class exhibited remarkably better outcomes compared to the amyloid‐growth class across multiple measures.
- LC analysis proves valuable in testing amyloid reduction as a surrogate biomarker in clinical trials lacking significant treatment effects.

## 1 BACKGROUND

Accumulation of amyloid beta (Aβ) species in the brain is a primary pathological feature of Alzheimer's disease (AD), believed to trigger downstream pathologies, neurodegeneration, and cognitive decline.^1^, ^2^, ^3^ There is a growing body of evidence supporting the notion that amyloid reduction could serve as a surrogate endpoint for clinical benefits.^4^ Both the phase 3 lecanemab and donanemab trials led to robust amyloid removal, and significant treatment effects across multiple clinical and cognitive endpoints^5^, ^6^ whereas in two identically designed phase 3 trials of gantenerumab with only partial amyloid removal, consistent treatment effects favoring gantenerumab were seen, but these effects were minimal and did not achieve significance in their primary endpoint.^7^ While these seemingly diverse treatment effects can be attributed to factors such as the rate and degree of amyloid removal associated with each drug,^8^ they have provided discussion and controversy regarding the amyloid hypothesis, with issues including understanding why some trials targeting Aβ have not shown efficacy,^9^, ^10^ and understanding the relationship between degree of cerebral amyloid reduction on positron emission tomography (PET) imaging and degree of clinical efficacy.^11^ Nonetheless, these diverse findings underscore the need for a comprehensive evaluation of amyloid PET reduction as a surrogate endpoint for clinical and cognitive benefits.

A surrogate endpoint is a measure used in clinical trials as a substitute for a direct assessment of core therapeutic targets such as a patient's well‐being, functional status, or survival. Its primary purpose is to predict the clinical benefit, rather than serving as a direct measure of that benefit. Typically, additional clinical trials are required to demonstrate that the surrogate endpoint reliably predicts or correlates with the desired clinical outcomes. The successful clinical trials of the anti‐amyloid treatments, lecanemab and donanemab, provided strong validation that significant reduction of brain amyloid plaque to normal levels is associated with a decrease in clinical decline over 18 months, making measures of amyloid plaques a potential surrogate biomarker. Our goal is to further validate this surrogate biomarker using clinical trials with small or non‐significant overall effects in clinical or cognitive endpoints.

Traditional validation of a surrogate endpoint involves estimating the correlation between biomarker changes and clinical/cognitive endpoints.^11^ While intuitive, this approach may overlook crucial differences in disease stages and treatment effects. Participants at different disease stages may exhibit varying clinical benefits, even with similar treatment effects on amyloid biomarkers. For example, a reduction of 20 Centiloid from baseline might mean a different treatment effect for a baseline Centiloid of 40 (early disease stage) versus a baseline of 90 (late disease stage) although it is treated the same in the correlation estimation. Alternative approaches that can distinguish these subtle differences, like latent class (LC) analysis,^12^ may offer a better chance to validate amyloid reduction as a surrogate biomarker in clinical trials without significant clinical benefits. LC analysis aims to identify distinct subgroups with different properties that may impact the clinical treatment effects. LC analysis does not require prior specification of these properties; instead, it only requires determining the number of classes to be identified beforehand. In the context of AD, these properties could represent disease stages (e.g., characterized by varying mean baseline amyloid standardized uptake value ratio [SUVR] values) or different patterns of disease progression with or without drug intervention (e.g., distinct annual rates of change in amyloid SUVR).

Our research hypothesis is that amyloid lowering during treatment is associated with slowed clinical and cognitive decline. Amyloid changes can manifest in three distinct ways: reduction from baseline, growth from baseline, or no significant change (flat) from baseline. Guided by this rationale, we pre‐specified three classes to be identified in the LC analysis. By using LC analysis, we aim to understand the relationship between amyloid reduction and clinical/cognitive benefits using data from the Dominantly Inherited Alzheimer Network Trials Unit (DIAN‐TU)−001 trial. Noticeably, findings from these post hoc analyses are descriptive in nature and should be interpreted in terms of what is clinically meaningful rather than based on statistical significance.

**RESEARCH IN CONTEXT**

1. **Systematic review**: The authors conducted a comprehensive literature review using both traditional sources like PubMed, as well as abstracts and presentations from scientific meetings. They identified and appropriately cited relevant publications related to the establishment of surrogate biomarkers for Alzheimer's disease (AD) clinical trials.
2. **Interpretation**: Accumulating evidence suggests that the reduction of amyloid plaques could serve as a credible surrogate endpoint for assessing clinical and cognitive efficacy in AD research. Latent class (LC) analysis classified participants in the Dominantly Inherited Alzheimer Network Trials Unit study into three distinct groups based on their longitudinal amyloid Pittsburgh compound B positron emission tomography standardized uptake value ratio data: those with no change in amyloid levels, those with amyloid reduction, and those with amyloid growth. Remarkably, despite similar baseline characteristics, the group with amyloid reduction exhibited a reduction in the annual decline rates compared to the group with amyloid growth across a spectrum of biomarkers, clinical parameters, and cognitive assessments. These findings derived from the LC analysis strongly support the notion that amyloid reduction is closely associated with improved clinical outcomes.
3. **Future directions**: If the LC analysis results can be validated in trials with larger sample sizes, it could play a crucial role in validating a surrogate biomarker. This validation, in turn, could significantly expedite the development of therapies for anti‐amyloid drugs.

## 2 METHODS

### 2.1 Study oversight

The DIAN‐TU study was conducted in accordance with the Declaration of Helsinki and the International Council for Harmonization and Good Clinical Practice guidelines and had ethics committee approval at each participating site. All participants provided written informed consent.

### 2.2 Study participants

The DIAN‐TU‐001 trial is a multicenter study, randomized, double‐blind, placebo‐controlled, cognitive endpoint, phase 2/3 trial that investigates potential disease‐modifying therapies (gantenerumab and solanezumab) in individuals with dominantly inherited AD mutations. The trial enrolled a total of 193 participants, including 144 mutation carriers (MCs) and 49 non–mutation carriers (NMCs). Further information regarding the study participants can be found in previous publications.^13^, ^14^ These participants were either cognitively normal (Clinical Dementia Rating [CDR = 0]) or exhibited early‐stage disease (CDR 0.5 or 1, representing very mild or mild dementia) at the time of enrollment. The trial used a common‐close design, ensuring that the double‐blind treatment continued for all participants (unless they dropped out early) until the last enrolled participant reached 4 years. All data collected during the follow‐up period were used in this report.

### 2.3 Clinical, cognitive, imaging, and cerebrospinal fluid biomarker outcomes

The analyzed clinical outcomes encompassed the CDR–Sum of Boxes^15^ (CDR‐SB) and Functional Assessment Scale (FAS).^16^ Cognitive outcomes included the Mini‐Mental State Examination (MMSE),^17^ the Wechsler Memory Scale‐Revised Logical Memory Delayed Recall Test (Logical Memory),^18^ the Wechsler Adult Intelligence Scale Digit Symbol Substitution Test (Digit Symbol),^18^ and the International Shopping List Test (ISLT) Delayed Recall score.^19^, ^20^ Imaging and cerebrospinal fluid (CSF) biomarker outcomes consisted of Pittsburgh compound B (PiB)‐PET standardized uptake value ratio (SUVR) composite, fluorodeoxyglucose (FDG)‐PET SUVR composite, magnetic resonance imaging (MRI)‐derived volumetrics, CSF total tau, CSF phosphorylated tau (p‐tau)181, and CSF neurofilament light (NfL). The methods used for processing imaging and CSF biomarkers have been previously described.^21^, ^22^, ^23^ All study personnel (including CDR‐SB and FAS raters), sponsors, and participants were blinded to the active or placebo assignment but not to the study drug arm.

### 2.4 Statistical analysis plan

To classify all participants into three groups based on amyloid change (amyloid reduction from baseline, amyloid growth from baseline, or amyloid no significant change from baseline), we used a linear mixed effects (LME) LC model. This model leverages both the baseline amyloid level and the post‐baseline amyloid reduction, offering distinct advantages. Each underlying class in the LME LC model follows a different LME model with distinct intercepts and slopes that will be estimated by the LC model. Participant classification is determined based on the individual probability of belonging to each class. To identify these underlying classes, we used longitudinal data from amyloid PiB PET SUVR, which is a widely used standard amyloid biomarker in clinical trials.

After the identification of the three underlying classes, we reported their baseline characteristics. For amyloid biomarkers, downstream biomarkers, and clinical/cognitive endpoints, we presented both the baseline mean and the longitudinal annual rate of change.

All analyses were performed using SAS software, version 9.4. Two‐sided *t* tests with a type I error of 0.05 were used, and nominal *P* values were reported. Confidence intervals (CIs) were presented as 95% CIs.[Table alz13735-tbl-0001], [Table alz13735-tbl-0002]

## 3 RESULTS

### 3.1 Latent classification and baseline characteristics

The LC analysis included a total of 139 participants with longitudinal PiB PET data. Among them, 24 participants (17.3%) were classified as amyloid reduction, 68 participants (48.9%) as amyloid growth, and 47 participants (33.8%) were classified as being amyloid no change (Table 1). Twenty‐three of 24 in the amyloid reduction group were from the treated arms, indicating the effectiveness of the treatment in reducing amyloid for this subset. However, one participant from the placebo group exhibited a negative rate of change in amyloid during the follow‐up and was classified by the model as amyloid reduction.

**TABLE 1 alz13735-tbl-0001:** Distribution of each treatment arm by the three latent classes.

	Latent classes			
Treatment arm	Amyloid no change (*N* = 47)	Amyloid reduction (*N* = 24)	Amyloid growth (*N* = 68)	Total
**Gantenerumab**	18 (34.6%)	20 (38.5%)	14 (26.9%)	52
**Placebo**	11 (27.5%)	1 (2.5%)	28 (70.0%)	40
**Solanezumab**	18 (38.3%)	3 (6.4%)	26 (55.3%)	47

The amyloid‐reduction class and the amyloid‐growth class demonstrate comparable baseline characteristics, as shown in Table 2, suggesting a balanced representation. Both classes appear to be in a more advanced disease stage compared to the amyloid‐no‐change class, as evidenced by their amyloid burden and clinical characteristics (Table 2).

**TABLE 2 alz13735-tbl-0002:** Baseline characteristics for the three latent classes.

Outcomes	Amyloid no change (*N* = 47)	Amyloid reduction (*N* = 24)	Amyloid growth (*N* = 68)	*P* value^*^
**CDR = 0, N (%)**	40 (85%)	12 (50%)	30 (44%)	0.62
**CDR > 0, N (%)**	7 (15%)	12 (50%)	38 (56%)	
**Digit symbol**	54.19 ± 17.87	40.75 ± 19.90	43.47 ± 19.69	0.55
**MMSE**	27.91 ± 3.44	26.88 ± 3.65	26.21 ± 3.95	0.45
**Logical memory**	12.09 ± 5.06	8.63 ± 6.70	8.71 ± 6.87	0.96
**International shopping list**	7.72 ± 3.47	5.17 ± 3.55	5.37 ± 4.41	0.83
**CDR‐SB**	0.72 ± 1.81	1.54 ± 2.04	1.71 ± 1.96	0.72
**PiB PET SUVR**	1.51 ± 0.47	3.23 ± 0.96	3.28 ± 1.15	0.82
**EYO**	−5.8 (7.5)	−0.22 (7.1)	−0.87 (6.2)	0.69

Abbreviations: CDR, Clinical Dementia Rating; CDR‐SB, Clinical Dementia Rating Sum of Boxes; EYO, estimated years from symptom onset; MMSE, Mini‐Mental State Examination; PET, positron emission tomography; PiB, Pittsburgh compound B; SUVR, standardized uptake value ratio.

* *P* values are for the comparison between amyloid‐reduction class and amyloid‐growth class only.

### 3.2 Comparison of amyloid reduction and downstream biomarker progression by latent classes

**FIGURE 1:**
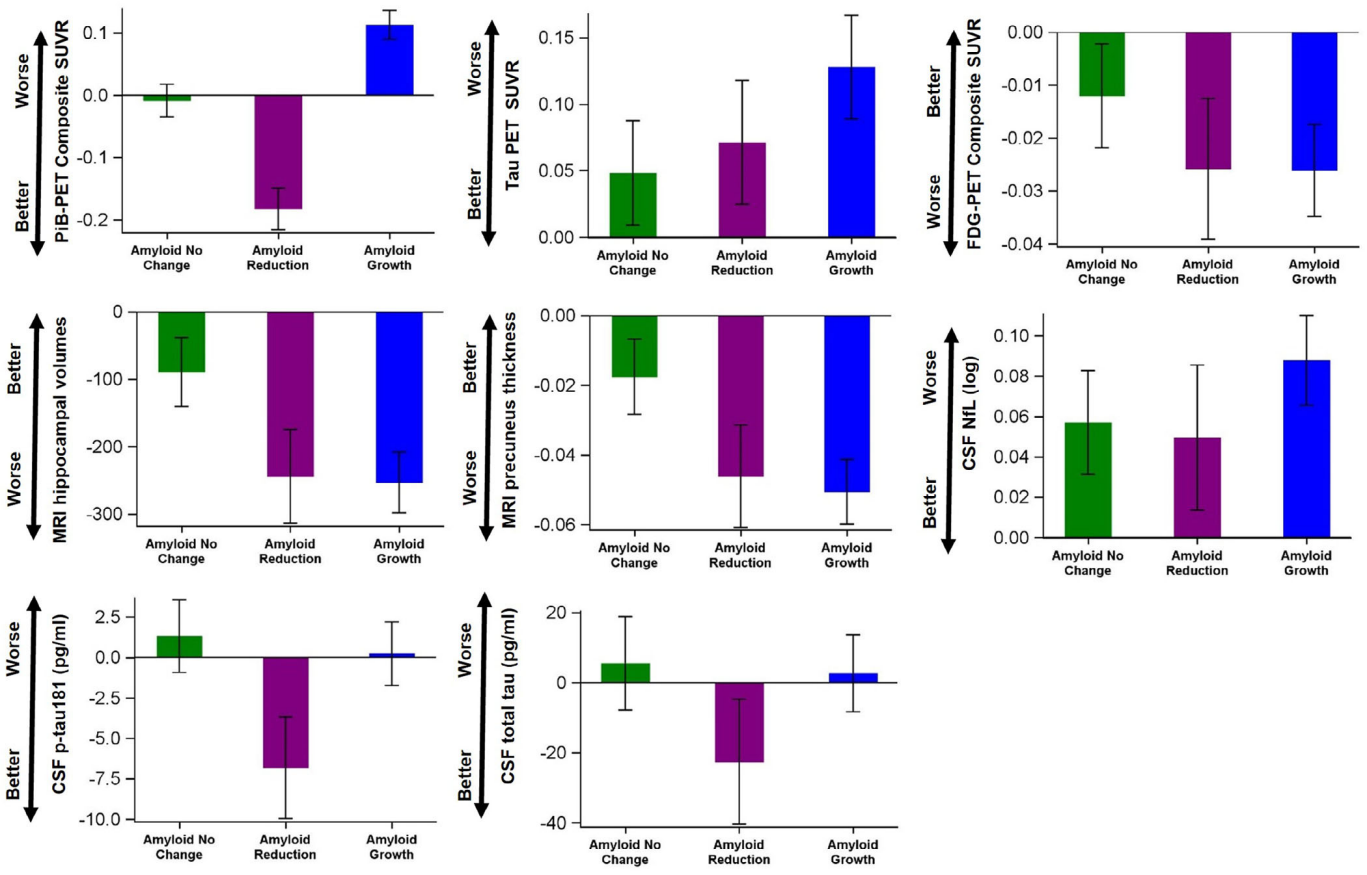
Estimated annual rate of change and 95% confidence interval (CI) by latent classes. 𝙸 bars (i.e., 95% CI) covering 0 indicate a non‐significant rate of change at a two‐sided type I error of 0.05. CSF, cerebrospinal fluid; MRI, magnetic resonance imaging; PET, positron emission tomography; PiB, Pittsburgh compound B; p‐tau, phosphorylated tau; SUVR, standardized uptake value ratio.

Figures 1 and S1 in supporting information illustrate the estimated annual rates of change for PiB PET SUVR and other downstream imaging and CSF biomarkers. Because PiB PET SUVR was used in the LC model, it was anticipated to demonstrate varying rates of change across the three classes. Consistent with the classification, the amyloid‐no‐change class showed a minimal rate of change during the follow‐up period. The amyloid‐reduction class exhibited a noteworthy negative rate of change, indicating a reduction in amyloid. On the other hand, the amyloid‐growth class demonstrated a significant positive rate of change, indicating substantial amyloid accumulation.

For tau PET composite SUVR, participants in the amyloid‐no‐change class exhibited slower rates of annual change compared to those in the amyloid‐reduction class who, in turn, demonstrated slower rates than those in the amyloid‐growth class (Table S1 in supporting information). Similar patterns were observed for MRI hippocampal volumes and MRI precuneus thickness although the differences between the amyloid‐reduction class and the amyloid‐growth class were much smaller (Table S1). Conversely, for FDG‐PET composite SUVR, both the amyloid‐reduction class and the amyloid‐growth class exhibited comparable rates of decline (Table S1). In terms of CSF NfL, numerically the smallest progression was observed among the amyloid‐reduction class. Furthermore, only the amyloid‐reduction class displayed significant reductions in CSF p‐tau181 and CSF total tau (Figures 1 and S1), highlighting the association of these biomarkers with amyloid PET reduction.

A sensitivity analysis was conducted, excluding the single placebo participant who was classified into the amyloid‐reduction class, and the results were found to be consistent (Figure S1).

### 3.3 Comparison of clinical and cognitive progression by latent classes

**FIGURE 2:**
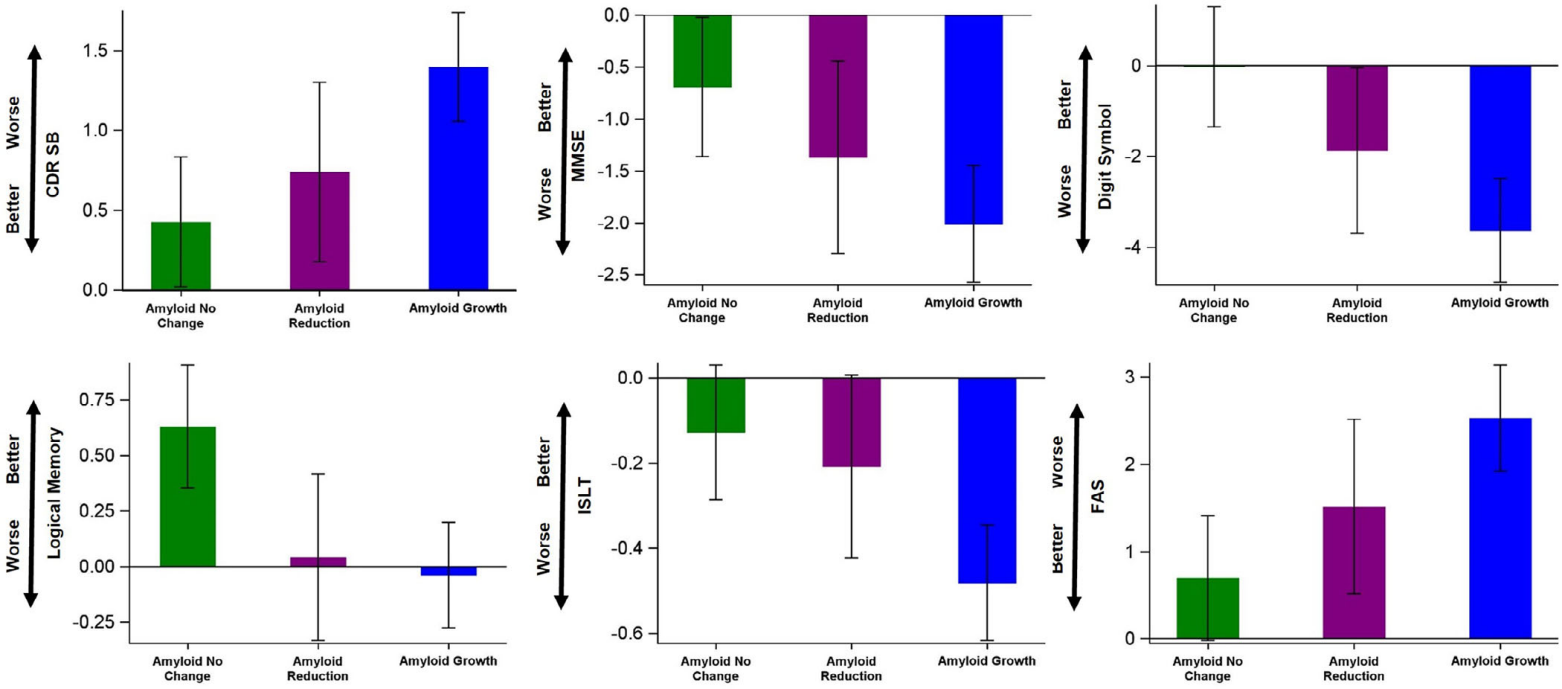
Estimated annual rate of change and 95% confidence interval (CI) by latent classes. 𝙸 bars (i.e., 95% CI) covering 0 indicate a non‐significant rate of change at a two‐sided type I error of 0.05. CDR SB, Clinical Dementia Rating Sum of Boxes; MMSE, Mini‐Mental State Examination; Digit Symbol, Digit Symbol Substitution Test; Logical Memory, logical memory delayed recall test; ISLT, International Shopping List Test‐Delayed Recall; FAS, Functional Assessment Scale.

Figures 2 and S2 in supporting information display the estimated annual rates of change for clinical and cognitive outcomes. Participants in the amyloid‐no‐change class showed an improvement in logical memory and displayed the least decline across all other outcome measures compared to the other two classes. Despite having similar baseline characteristics, the amyloid‐reduction class exhibited a reduction in the annual decline rate compared to the amyloid‐growth class across multiple measures: CDR‐SB decline was reduced by 47.1%, MMSE by 31.8%, Digit Symbol by 48.2%, ISLT by 56.3%, and FAS by 40.3% (Table S2 in supporting information). Three sets of sensitivity analyses were performed to examine the robustness of the potential association between amyloid reduction and clinical/cognitive benefits. These included the exclusion of the single placebo participant (Figure S2), the use of only the first 24 months of clinical/cognitive data (Figure S3 in supporting information), and the inclusion of additional key covariates (Figure S4 in supporting information). The results from these sensitivity analyses align with the main findings in this report.

## 4 DISCUSSION

The testing of amyloid reduction as a surrogate biomarker is crucial for enhancing our understanding of AD progression, as well as for guiding drug development and the regulatory process. The significant clinical decline slowing achieved by donanemab^6^, ^24^ and lecanemab^5^ provides strong evidence that amyloid reduction is associated with slower decline in cognition. Other clinical trials with anti‐amyloid drugs that did not fully remove amyloid did not reach statistical significance, although some exhibited small cognitive benefits in the expected direction.^13^, ^25^ This accumulating evidence presents an opportunity to better determine the amount of amyloid reduction that may serve as a surrogate biomarker and facilitate the acceleration of therapy development in this area.

In this context, we demonstrate how LC analysis can offer an alternative method of evaluating the effects of amyloid reduction on clinical and non‐amyloid biomarkers in clinical trials without significant clinical treatment effects of the primary outcome. By better distinguishing amyloid changes during the post‐baseline follow‐up, LC analysis enables the analysis of surrogate endpoints in clinical trials in which the primary analysis did not achieve significance, but evidence suggests a clear impact on the primary target. For example, in the DIAN‐TU trial, although no overall treatment effect was observed in cognitive outcomes and tau PET, significant amyloid reduction was observed in the gantenerumab‐treated group. The LC analysis successfully categorized participants into three subgroups based on their post‐baseline amyloid changes and baseline amyloid levels: amyloid no change, amyloid reduction, and amyloid growth. Subsequent comparisons among these subgroups provided a clearer understanding of the association between disease stage and amyloid reduction and the deceleration of cognitive decline and tau growth.

Notably, participants in the amyloid‐no‐change class demonstrated minimum amyloid accumulation and much less unfavorable progression in other biomarkers compared to the other two classes. Table 2 indicated that the amyloid‐no‐change class was in earlier disease stages than the other two classes, thus these findings underscore the influence of disease stages on the magnitude of amyloid change over time,^2^, ^26^ which can subsequently be modified by treatment intervention. Furthermore, the amyloid‐no‐change class experienced practice effect in logical memory test and exhibited less decline in the other clinical and cognitive outcomes (Figure 2) compared to those in later stages (amyloid reduction and amyloid growth classes). This is again likely due to the fact that the amyloid‐no‐change class was in an earlier disease stage compared to the other two classes, as indicated by the baseline amyloid burden, clinical scores, and cognitive scores (see Table 2 for baseline comparison) and learned during the trial.^27^, ^28^ Moreover, among the more advanced participants, those belonging to the amyloid‐reduction class demonstrated a remarkable reduction of > 30% in the annual clinical decline rate across five cognitive outcomes compared to those in the amyloid‐growth class. Interestingly, these reductions are in line with the magnitudes observed with donanemab^6^, ^24^ and lecanemab.^5^ These findings reinforce the importance of considering the rate and extent of amyloid removal in the context of negative clinical trials.^29^, ^30^ They provide additional support for the amyloid hypothesis and further validate amyloid reduction as a potential surrogate biomarker. As an additional point, it is worth mentioning that while we used amyloid PET as the outcome measure to determine the latent classes, other amyloid biomarkers such as CSF Aβ42 can also be used. However, in the DIAN‐TU trial, CSF Aβ42 was analyzed using different assays for participants treated with gantenerumab and solanezumab. Consequently, it is not possible to pool the results and use CSF Aβ42 as the outcome measure to determine the latent classes. Future work will examine other biomarkers, including p‐tau.

There are some limitations of the LC analysis. First, the model‐determined classes are not randomized, which may yield less robust statistical inference compared to randomized clinical trials. Second, the constrained sample size of the DIAN‐TU study limits the complexity of the underlying model in the LC analysis. Larger trials can explore other longitudinal models with more parameters.^31^, ^32^, ^33^ Last, the LC analysis necessitates pre‐specifying the number of classes to be identified by the model, which should be determined based on scientific judgment and the overall sample size.

In summary, LC analysis harnesses the comprehensive profiles of individuals to categorize participants based on response to treatment, making it an informative tool for establishing associations between variables or identifying subgroups that may benefit from treatment, even when the overall trial does not demonstrate a clinical treatment effect by randomization.

## CONFLICT OF INTEREST AND DISCLOSURE STATEMENT

Guoqiao Wang, PhD, is the biostatistics core co‐leader for the DIAN‐TU. He reports serving on a Data Safety Committee for Eli Lilly and Company and statistical consultant for Alector. Tammie L. S. Benzinger, M.D., Ph.D., has investigator‐initiated research funding from the NIH, the Alzheimer's Association, the Barnes‐Jewish Hospital Foundation, and Avid Radiopharmaceuticals. Dr. Benzinger participates as a site investigator in clinical trials sponsored by Avid Radiopharmaceuticals, Eli Lilly and Company, Biogen, Eisai, Jaansen, and F. Hoffmann‐La Roche, Ltd. She serves as an unpaid consultant to Eisai and Siemens. She is on the Speaker's Bureau for Biogen. Jason Hassenstab, Ph.D., is a paid consultant for F. Hoffmann‐La Roche, Ltd., Takeda, and Lundbeck, and is on the Data Safety and Monitoring Board for Eisai. Andrew J. Aschenbrenner, Ph.D., has served as a consultant for Biogen Inc., and H. Lundbeck HS. Eric McDade, D.O., is the Associate Director of the DIAN‐TU. He reports serving on a Data Safety Committee for Eli Lilly and Company and Alector; as a scientific consultant for Eisai and Eli Lilly and Company; receiving institutional grant support from Eli Lilly and Company, F. Hoffmann‐La Roche, Ltd., and Janssen. David B. Clifford, M.D., is Medical Director of the DIAN‐TU and serves as scientific consultant to Biogen, Takeda, Millennium, Genzyme, Amgen, F. Hoffmann‐La Roche, Ltd./Genentech, Glaxo Smith Kline, Serono, Inhibikase, Dr Reddy's Lab, Bristol Myers Squibb, Atara, Mitsubishi Tanabe, Excision BioTherapeutics, Up to Date, and Wolters Kluwer; on DSMB/Data Monitoring Committees for Genentech/F. Hoffmann‐La Roche, Ltd., Wave, EMD Serono, Shire, Pfizer, Sanofi; performing legal consulting: Cook County, State Farm, Wilke & Wilke PC, Shevlin Smith, Sal Indomenico PC; and receiving research support from NIH NINDS, NIMH, NIAID, NCATS and NIA. Gregg Day's research is supported by NIH (K23AG064029, U01AG057195, U01NS120901, U19AG032438), the Alzheimer's Association, and Chan Zuckerberg Initiative. He serves as a consultant for Parabon Nanolabs Inc., as a Topic Editor (*Dementia*) for DynaMed (EBSCO), and as the Clinical Director of the Anti‐NMDA Receptor Encephalitis Foundation (Inc, Canada; uncompensated). He is the co‐Project PI for a clinical trial in anti‐NMDAR encephalitis, which receives support from Horizon Pharmaceuticals. He has developed educational materials for PeerView Media, Inc., and Continuing Education Inc. He owns stock in ANI pharmaceuticals. Dr. Day's institution has received support from Eli Lilly for Dr. Day's development and participation in an educational event promoting early diagnosis of symptomatic Alzheimer's disease, and in‐kind contributions of radiotracer precursors (via Avid Radiopharmaceuticals, a wholly owned subsidiary of Eli Lilly). John M. Ringman receives support from Avid by provision of flortaucipir for his studies. Tobias Bittner is a full‐time employee of F. Hoffmann‐LaRoche Ltd. and Genentech Inc., a member of the Roche Group, and shareholder of F. Hoffmann‐LaRoche Ltd. Randall J. Bateman, M.D., is the Director of the DIAN‐TU and Principal Investigator of the DIAN‐TU‐001. He receives research support from the National Institute on Aging of the National Institutes of Health, DIAN‐TU Trial Pharmaceutical Partners (Eli Lilly and Company, F. Hoffman‐La Roche, Ltd., and Avid Radiopharmaceuticals), Alzheimer's Association, GHR Foundation, Anonymous Organization, DIAN‐TU Pharma Consortium (Active: Biogen, Eisai, Eli Lilly and Company, Janssen, F. Hoffmann‐La Roche, Ltd./Genentech and previously from: AbbVie, Amgen, AstraZeneca, Forum, Mithridion, Novartis, Pfizer, Sanofi, United Neuroscience). He has been an invited speaker for Novartis and serves on the Advisory Board for F. Hoffman La Roche, Ltd.

Catherine J. Mummery receives support from the NIHR UCLH Biomedical Research Centre. She has consulted for Biogen, Eli Lilly and Company, Eisai, IONIS, WAVE, F. Hoffman‐La Roche Ltd, Prevail, Alector, and Immunobrain. Nick C. Fox reports consulting fees from Biogen, Eisai, Ionis, Lilly, Roche/Genentech, and Siemens, all paid to UCL; he has served on a Data Safety Monitoring Board for Biogen; he acknowledges grant support from the Alzheimer's Society, Alzheimer's Research UK, Rosetrees Trust, the UK Dementia Research Institute, the UK NIHR UCLH Biomedical Research Centre. All the other authors reported no conflicts of interest. Author disclosures are available in the supporting information.

## CONSENT STATEMENT

The DIAN‐TU study was conducted in accordance with the Declaration of Helsinki and the International Council for Harmonization and Good Clinical Practice guidelines and had ethics committee approval at each participating site. All participants provided written informed consent.

## Supporting information

Supporting Information

ICJME coi disclosure

Supporting Information

## References

[1] Bateman RJ , Xiong C , Benzinger TL , et al. Clinical and biomarker changes in dominantly inherited Alzheimer's disease. N Engl J Med. 2012;367(9):795‐804. 22784036 10.1056/NEJMoa1202753 PMC3474597

[2] Wang G , Coble D , McDade EM , et al. Staging biomarkers in preclinical autosomal dominant Alzheimer's disease by estimated years to symptom onset. Alzheimers Dement. 2019;15(4):506‐514. 30773445 10.1016/j.jalz.2018.12.008 PMC6461496

[3] Salloway S , Farlow M , McDade E , et al. A trial of gantenerumab or solanezumab in dominantly inherited Alzheimer's disease. Nat Med. 2021;27(7):1187‐1196. 34155411 10.1038/s41591-021-01369-8 PMC8988051

[4] Dunn B , Stein P , Temple R , Cavazzoni P . An appropriate use of accelerated approval—Aducanumab for Alzheimer's disease. N Engl J Med. 2021;385(9):856‐857. 34320283 10.1056/NEJMc2111960

[5] van Dyck CH , Swanson CJ , Aisen P , et al. Lecanemab in early Alzheimer's disease. N Engl J Med. 2023;388(1):9‐21. 36449413 10.1056/NEJMoa2212948

[6] Sims JR , Zimmer JA , Evans CD , et al. Donanemab in early symptomatic Alzheimer disease: the TRAILBLAZER‐ALZ 2 randomized clinical trial. JAMA. 2023;330(6):512‐527. 37459141 10.1001/jama.2023.13239 PMC10352931

[7] Lane C , Smith J , Perry RJ , et al. GRADUATE I AND II: revisiting the key findings from two Phase III studies evaluating the efficacy and safety of subcutaneous gantenerumab in early Alzheimer's disease (AD). Paper presented at: Alzheimer's Association International Conference 2023.

[8] Hardy J , Mummery C . An anti‐amyloid therapy works for Alzheimer's disease: why has it taken so long and what is next? Brain. 2023;146(4):1240‐1242. 36797987 10.1093/brain/awad049 PMC10115350

[9] Makin S . The amyloid hypothesis on trial. Nature. 2018;559(7715):S4‐S4. 30046080 10.1038/d41586-018-05719-4

[10] Honig LS . Alzheimer disease: a new beginning in therapeutics. In. Vol 37: LWW; 2023:267‐269. 10.1097/WAD.0000000000000592 38015422

[11] Budd Haeberlein S , Aisen P , Barkhof F , et al. Two randomized phase 3 studies of aducanumab in early Alzheimer's disease. J Prev Alzheimer's Dis. 2022;9(2):197‐210. 35542991 10.14283/jpad.2022.30

[12] McCutcheon AL . Latent class analysis. Sage; 1987.

[13] Salloway S , Farlow M , McDade E , et al. A trial of gantenerumab or solanezumab in dominantly inherited Alzheimer's disease. Nat Med. 2021;27(7):1187‐1196. 34155411 10.1038/s41591-021-01369-8 PMC8988051

[14] Wang G , Li Y , Xiong C , et al. Evaluation of dose‐dependent treatment effects after mid‐trial dose escalation in biomarker, clinical, and cognitive outcomes for gantenerumab or solanezumab in dominantly inherited Alzheimer's disease. Alzheimers Dement. 2022;14(1):e12367. 10.1002/dad2.12367 PMC9632865 36348972

[15] Berg L , Miller JP , Storandt M , et al. Mild senile dementia of the Alzheimer type: 2. Longitudinal assessment. Ann Neurol. 1988;23(5):477‐484. 3389756 10.1002/ana.410230509

[16] Breines E . The functional assessment scale as an instrument for measuring changes in levels of function of nursing home residents following occupational therapy. Can J Occup Ther. 1988;55(3):135‐140.

[17] Folstein M . A practical method for grading the cognitive state of patients for the children. J Psychiatr Res. 1975;12:189‐198. 1202204 10.1016/0022-3956(75)90026-6

[18] Dumont R , Willis JO , Veizel K , Zibulsky J . Wechsler Adult Intelligence Scale–Fourth Edition. Encyclopedia of Special Education: A Reference for the Education of Children, Adolescents, and Adults with Disabilities and Other Exceptional Individuals . 2013.

[19] Lim YY , Prang KH , Cysique L , Pietrzak RH , Snyder PJ , Maruff P . A method for cross‐cultural adaptation of a verbal memory assessment. Behav Res Methods. 2009;41(4):1190‐1200. 19897828 10.3758/BRM.41.4.1190

[20] Thompson TA , Wilson PH , Snyder PJ , et al. Sensitivity and test–retest reliability of the international shopping list test in assessing verbal learning and memory in mild Alzheimer's disease. Arch Clin Neuropsychol. 2011;26(5):412‐424. 21613302 10.1093/arclin/acr039

[21] Bateman RJ , Xiong C , Benzinger TL , et al. Clinical and biomarker changes in dominantly inherited Alzheimer's disease. N Engl J Med. 2012;367:795‐804. 22784036 10.1056/NEJMoa1202753 PMC3474597

[22] Benzinger TL , Blazey T , Jack CR , et al. Regional variability of imaging biomarkers in autosomal dominant Alzheimer's disease. Proc Natl Acad Sci. 2013;110(47):E4502‐E4509. 24194552 10.1073/pnas.1317918110 PMC3839740

[23] Su Y , Blazey TM , Snyder AZ , et al. Partial volume correction in quantitative amyloid imaging. Neuroimage. 2015;107:55‐64. 25485714 10.1016/j.neuroimage.2014.11.058 PMC4300252

[24] Mintun MA , Lo AC , Duggan Evans C , et al. Donanemab in early Alzheimer's disease. N Engl J Med. 2021;384(18):1691‐1704. 33720637 10.1056/NEJMoa2100708

[25] Smith J , Donohue MC , Gruendl E , et al. GRADUATE I AND II: findings of two phase III randomized placebo‐controlled studies assessing the efficacy and safety of subcutaneous gantenerumab in early Alzheimer's disease (AD)(S26. 010). In: AAN Enterprises; 2023.

[26] McDade E , Wang G , Gordon BA , et al. Longitudinal cognitive and biomarker changes in dominantly inherited Alzheimer disease. Neurology. 2018;91(14):e1295‐e1306. 30217935 10.1212/WNL.0000000000006277 PMC6177272

[27] Wang G , Kennedy RE , Goldberg TE , Fowler ME , Cutter GR , Schneider LS . Using practice effects for targeted trials or sub‐group analysis in Alzheimer's disease: how practice effects predict change over time. PLoS One. 2020;15(2):e0228064. 32084191 10.1371/journal.pone.0228064 PMC7034859

[28] Aschenbrenner AJ , Hassenstab J , Wang G , et al. Avoid or embrace? practice effects in Alzheimer's disease prevention trials. Front Aging Neurosci. 2022;14:883131. 35783127 10.3389/fnagi.2022.883131 PMC9244171

[29] Rother C , Uhlmann RE , Müller SA , et al. Experimental evidence for temporal uncoupling of brain Aβ deposition and neurodegenerative sequelae. Nat Commun. 2022;13(1):7333. 36443293 10.1038/s41467-022-34538-5 PMC9705543

[30] Karran E , De Strooper B . The amyloid hypothesis in Alzheimer disease: new insights from new therapeutics. Nat Rev Drug Discovery. 2022;21(4):306‐318. 35177833 10.1038/s41573-022-00391-w

[31] Wang G , Liu L , Li Y , et al. Proportional constrained longitudinal data analysis models for clinical trials in sporadic Alzheimer's disease. Alzheimers Dement. 2022;8(1):e12286. 10.1002/trc2.12286 PMC8984094 35415211

[32] Lu K . On efficiency of constrained longitudinal data analysis versus longitudinal analysis of covariance. Biometrics. 2010;66(3):891‐896. 19764951 10.1111/j.1541-0420.2009.01332.x

[33] Durrleman S , Simon R . Flexible regression models with cubic splines. Stat Med. 1989;8(5):551‐561. 2657958 10.1002/sim.4780080504

