# Supplementary material for: Examining amyloid reduction as a surrogate endpoint through latent class analysis using clinical trial data for dominantly inherited Alzheimer's disease
Source: Alzheimers Dement. 2024 Feb 23;20(4):2698–706. doi: 10.1002/alz.13735 (PMC11032558; doi:10.1002/alz.13735)
Supplement: Supplementary file 1 — Supporting Information [file ALZ-20-2698-s003.docx]

**Supplemental Table** **1**: Estimated baseline means, annual rates of change and their comparison (**Bold rows**) for biomarker outcomes by latent classes

| **Outcome** | **Label** | **Estimate** | **SE** | **P-value** | **95% CI Lower**  **Bound** | **95% CI Upper**  **Bound** |
| --- | --- | --- | --- | --- | --- | --- |
| **PiB-PET Composite SUVR** | Baseline mean for Amyloid No Change class | 1.542 | 0.136 | <.0001 | 1.2733 | 1.8102 |
|  | Baseline mean for Amyloid Reduction class | 3.189 | 0.190 | <.0001 | 2.8134 | 3.5645 |
|  | Baseline mean for Amyloid Growth class | 3.260 | 0.113 | <.0001 | 3.0371 | 3.4835 |
|  | Annual rate of change for Amyloid No Change class | -0.009 | 0.013 | 0.5217 | -0.0347 | 0.01767 |
|  | Annual rate of change for Amyloid Reduction class | -0.183 | 0.017 | <.0001 | -0.2163 | -0.1491 |
|  | Annual rate of change for Amyloid Growth class | 0.113 | 0.012 | <.0001 | 0.08998 | 0.1362 |
|  | **Amyloid Reduction vs Amyloid Growth** | **-0.296** | **0.021** | **<.0001** | **-0.3366** | **-0.255** |
|  | **Amyloid No Change vs Amyloid Reduction** | **0.174** | **0.022** | **<.0001** | **0.1316** | **0.2168** |
|  | **Amyloid No Change vs Amyloid Growth** | **-0.122** | **0.018** | **<.0001** | **-0.1566** | **-0.0867** |
| **Tau PET SUVR** | Baseline mean for Amyloid No Change class | 1.461 | 0.158 | <.0001 | 1.1435 | 1.7792 |
|  | Baseline mean for Amyloid Reduction class | 1.849 | 0.220 | <.0001 | 1.4073 | 2.2915 |
|  | Baseline mean for Amyloid Growth class | 2.145 | 0.138 | <.0001 | 1.8662 | 2.4229 |
|  | Annual rate of change for Amyloid No Change class | 0.048 | 0.019 | 0.0169 | 0.00908 | 0.08749 |
|  | Annual rate of change for Amyloid Reduction class | 0.071 | 0.023 | 0.0035 | 0.02468 | 0.1177 |
|  | Annual rate of change for Amyloid Growth class | 0.128 | 0.019 | <.0001 | 0.08893 | 0.1666 |
|  | **Amyloid Reduction vs Amyloid Growth** | **-0.057** | **0.030** | **0.0664** | **-0.1172** | **0.00398** |
|  | **Amyloid No Change vs Amyloid Reduction** | **-0.023** | **0.030** | **0.4527** | **-0.0837** | **0.03792** |
|  | **Amyloid No Change vs Amyloid Growth** | **-0.080** | **0.027** | **0.0057** | **-0.1347** | **-0.0243** |
| **FDG-PET Composite SUVR** | Baseline mean for Amyloid No Change class | 1.667 | 0.023 | <.0001 | 1.6226 | 1.7122 |
|  | Baseline mean for Amyloid Reduction class | 1.637 | 0.031 | <.0001 | 1.5745 | 1.6987 |
|  | Baseline mean for Amyloid Growth class | 1.622 | 0.019 | <.0001 | 1.585 | 1.659 |
|  | Annual rate of change for Amyloid No Change class | -0.012 | 0.005 | 0.0168 | -0.0218 | -0.0022 |
|  | Annual rate of change for Amyloid Reduction class | -0.026 | 0.007 | 0.0002 | -0.0391 | -0.0125 |
|  | Annual rate of change for Amyloid Growth class | -0.026 | 0.004 | <.0001 | -0.0349 | -0.0174 |
|  | **Amyloid Reduction vs Amyloid Growth** | **0.000** | **0.008** | **0.9673** | **-0.0156** | **0.01623** |
|  | **Amyloid No Change vs Amyloid Reduction** | **0.014** | **0.008** | **0.1013** | **-0.0027** | **0.03033** |
|  | **Amyloid No Change vs Amyloid Growth** | **0.014** | **0.007** | **0.0351** | **0.001** | **0.02726** |
| **MRI hippocampal volumes** | Baseline mean for Amyloid No Change class | 8748 | 173 | <.0001 | 8408 | 9088 |
|  | Baseline mean for Amyloid Reduction class | 7972 | 242 | <.0001 | 7495 | 8448 |
|  | Baseline mean for Amyloid Growth class | 7636 | 148 | <.0001 | 7344 | 7927 |
|  | Annual rate of change for Amyloid No Change class | -90 | 26 | 0.0007 | -141 | -38 |
|  | Annual rate of change for Amyloid Reduction class | -244 | 35 | <.0001 | -314 | -175 |
|  | Annual rate of change for Amyloid Growth class | -253 | 23 | <.0001 | -298 | -208 |
|  | **Amyloid Reduction vs Amyloid Growth** | **9** | **42** | **0.8317** | **-74** | **92** |
|  | **Amyloid No Change vs Amyloid Reduction** | **155** | **44** | **0.0005** | **68** | **241** |
|  | **Amyloid No Change vs Amyloid Growth** | **164** | **35** | **<.0001** | **95** | **232** |
| **MRI precuneus thickness** | Baseline mean for Amyloid No Change class | 2.302 | 0.029 | <.0001 | 2.2454 | 2.3578 |
|  | Baseline mean for Amyloid Reduction class | 2.225 | 0.040 | <.0001 | 2.1457 | 2.3032 |
|  | Baseline mean for Amyloid Growth class | 2.186 | 0.024 | <.0001 | 2.139 | 2.2327 |
|  | Annual rate of change for Amyloid No Change class | -0.018 | 0.005 | 0.0015 | -0.0283 | -0.0068 |
|  | Annual rate of change for Amyloid Reduction class | -0.046 | 0.007 | <.0001 | -0.0608 | -0.0314 |
|  | Annual rate of change for Amyloid Growth class | -0.051 | 0.005 | <.0001 | -0.0599 | -0.0413 |
|  | **Amyloid Reduction vs Amyloid Growth** | **0.005** | **0.009** | **0.6098** | **-0.0129** | **0.0219** |
|  | **Amyloid No Change vs Amyloid Reduction** | **0.029** | **0.009** | **0.0022** | **0.01031** | **0.04676** |
|  | **Amyloid No Change vs Amyloid Growth** | **0.033** | **0.007** | **<.0001** | **0.0188** | **0.0473** |
| **CSF NfL (log)** | Baseline mean for Amyloid No Change class | 6.270 | 0.088 | <.0001 | 6.0976 | 6.4427 |
|  | Baseline mean for Amyloid Reduction class | 6.793 | 0.125 | <.0001 | 6.5458 | 7.0392 |
|  | Baseline mean for Amyloid Growth class | 6.669 | 0.073 | <.0001 | 6.5252 | 6.8123 |
|  | Annual rate of change for Amyloid No Change class | 0.057 | 0.013 | <.0001 | 0.03134 | 0.08269 |
|  | Annual rate of change for Amyloid Reduction class | 0.049 | 0.018 | 0.0073 | 0.01349 | 0.08542 |
|  | Annual rate of change for Amyloid Growth class | 0.088 | 0.011 | <.0001 | 0.06555 | 0.1099 |
|  | **Amyloid Reduction vs Amyloid Growth** | **-0.038** | **0.021** | **0.0756** | **-0.0805** | **0.00398** |
|  | **Amyloid No Change vs Amyloid Reduction** | **0.008** | **0.022** | **0.7362** | **-0.0366** | **0.05175** |
|  | **Amyloid No Change vs Amyloid Growth** | **-0.031** | **0.017** | **0.0758** | **-0.0646** | **0.00322** |
| **CSF p-tau181 (pg/ml)** | Baseline mean for Amyloid No Change class | 67.527 | 9.370 | <.0001 | 49.0531 | 86.0005 |
|  | Baseline mean for Amyloid Reduction class | 97.498 | 13.393 | <.0001 | 71.0937 | 123.9 |
|  | Baseline mean for Amyloid Growth class | 112.490 | 7.847 | <.0001 | 97.0151 | 127.96 |
|  | Annual rate of change for Amyloid No Change class | 1.317 | 1.142 | 0.2502 | -0.9347 | 3.5683 |
|  | Annual rate of change for Amyloid Reduction class | -6.816 | 1.596 | <.0001 | -9.9631 | -3.669 |
|  | Annual rate of change for Amyloid Growth class | 0.227 | 0.993 | 0.8197 | -1.7312 | 2.1844 |
|  | **Amyloid Reduction vs Amyloid Growth** | **-7.043** | **1.880** | **0.0002** | **-10.749** | **-3.3364** |
|  | **Amyloid No Change vs Amyloid Reduction** | **8.133** | **1.963** | **<.0001** | **4.2633** | **12.0024** |
|  | **Amyloid No Change vs Amyloid Growth** | **1.090** | **1.513** | **0.4721** | **-1.8935** | **4.0739** |
| **CSF total tau** | Baseline mean for Amyloid No Change class | 406.610 | 49.190 | <.0001 | 309.59 | 503.64 |
|  | Baseline mean for Amyloid Reduction class | 513.380 | 67.744 | <.0001 | 379.76 | 647.01 |
|  | Baseline mean for Amyloid Growth class | 638.940 | 39.719 | <.0001 | 560.59 | 717.29 |
|  | Annual rate of change for Amyloid No Change class | 5.524 | 6.744 | 0.4138 | -7.7784 | 18.826 |
|  | Annual rate of change for Amyloid Reduction class | -22.561 | 9.039 | 0.0134 | -40.391 | -4.7303 |
|  | Annual rate of change for Amyloid Growth class | 2.635 | 5.565 | 0.6363 | -8.3414 | 13.6123 |
|  | **Amyloid Reduction vs Amyloid Growth** | **-25.196** | **10.615** | **0.0186** | **-46.134** | **-4.2577** |
|  | **Amyloid No Change vs Amyloid Reduction** | **28.084** | **11.278** | **0.0136** | **5.8387** | **50.3301** |
|  | **Amyloid No Change vs Amyloid Growth** | **2.888** | **8.743** | **0.7415** | **-14.358** | **20.1348** |


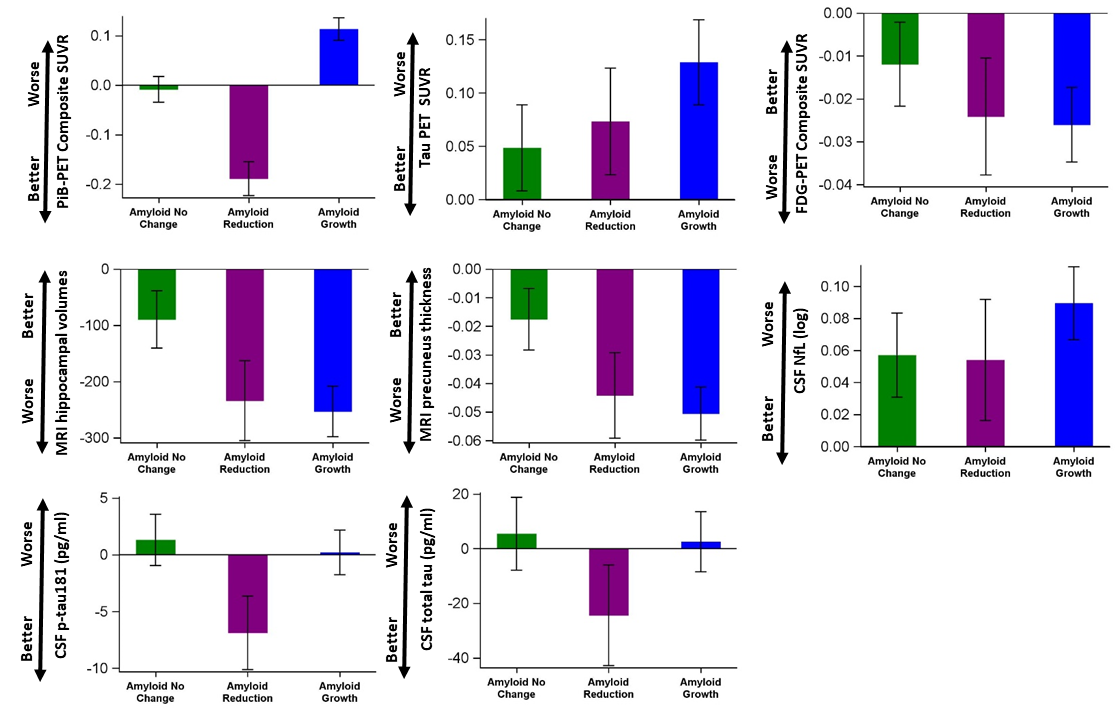


**Supplemental Figure** **1**: Estimated annual rate of change and 95% confidence interval (CI) by latent classes. 𝙸 bars (i.e., 95% CI) covering 0 indicate a non-significant rate of change at a two-sided type I error of 0.05. The single placebo participant in the amyloid-reduction class was excluded.

**Supplemental Table** **2**: Estimated baseline means, annual rates of change and their comparison (**Bold rows**) for clinical and cognitive outcomes by latent classes

| **Outcome** | **Label** | **Estimate** | **SE** | **P-value** | **95% CI Lower**  **Bound** | **95% CI Upper**  **Bound** |
| --- | --- | --- | --- | --- | --- | --- |
| CDR SB | Baseline mean for Amyloid No Change class | 0.68 | 0.30 | 0.0222 | 0.10 | 1.27 |
|  | Baseline mean for Amyloid Reduction class | 1.57 | 0.41 | 0.0002 | 0.75 | 2.38 |
|  | Baseline mean for Amyloid Growth class | 1.51 | 0.25 | <.0001 | 1.03 | 2.00 |
|  | Annual rate of change for Amyloid No Change class | 0.43 | 0.21 | 0.0409 | 0.02 | 0.83 |
|  | Annual rate of change for Amyloid Reduction class | 0.74 | 0.29 | 0.0102 | 0.18 | 1.30 |
|  | Annual rate of change for Amyloid Growth class | 1.40 | 0.17 | <.0001 | 1.05 | 1.74 |
|  | **Amyloid Reduction vs Amyloid Growth** | **-0.66** | **0.33** | **0.0499** | **-1.32** | **0.00** |
|  | **Amyloid No Change vs Amyloid Reduction** | **-0.31** | **0.35** | **0.3759** | **-1.01** | **0.38** |
|  | **Amyloid No Change vs Amyloid Growth** | **-0.97** | **0.27** | **0.0004** | **-1.50** | **-0.44** |
| MMSE | Baseline mean for Amyloid No Change class | 28.00 | 0.56 | <.0001 | 26.89 | 29.10 |
|  | Baseline mean for Amyloid Reduction class | 27.12 | 0.78 | <.0001 | 25.58 | 28.66 |
|  | Baseline mean for Amyloid Growth class | 26.53 | 0.47 | <.0001 | 25.62 | 27.45 |
|  | Annual rate of change for Amyloid No Change class | -0.69 | 0.34 | 0.0422 | -1.36 | -0.02 |
|  | Annual rate of change for Amyloid Reduction class | -1.37 | 0.47 | 0.0038 | -2.29 | -0.45 |
|  | Annual rate of change for Amyloid Growth class | -2.01 | 0.28 | <.0001 | -2.57 | -1.45 |
|  | **Amyloid Reduction vs Amyloid Growth** | **0.64** | **0.55** | **0.2438** | **-0.44** | **1.72** |
|  | **Amyloid No Change vs Amyloid Reduction** | **0.68** | **0.58** | **0.2451** | **-0.47** | **1.82** |
|  | **Amyloid No Change vs Amyloid Growth** | **1.32** | **0.44** | **0.0032** | **0.44** | **2.19** |
| Digit Symbol  Substitution Test | Baseline mean for Amyloid No Change class | 56.00 | 2.82 | <.0001 | 50.47 | 61.53 |
|  | Baseline mean for Amyloid Reduction class | 41.96 | 3.94 | <.0001 | 34.23 | 49.70 |
|  | Baseline mean for Amyloid Growth class | 45.58 | 2.34 | <.0001 | 40.98 | 50.18 |
|  | Annual rate of change for Amyloid No Change class | -0.03 | 0.68 | 0.9694 | -1.35 | 1.30 |
|  | Annual rate of change for Amyloid Reduction class | -1.88 | 0.93 | 0.0438 | -3.70 | -0.05 |
|  | Annual rate of change for Amyloid Growth class | -3.63 | 0.58 | <.0001 | -4.77 | -2.49 |
|  | **Amyloid Reduction vs Amyloid Growth** | **1.75** | **1.10** | **0.1096** | **-0.40** | **3.91** |
|  | **Amyloid No Change vs Amyloid Reduction** | **1.85** | **1.15** | **0.1077** | **-0.41** | **4.10** |
|  | **Amyloid No Change vs Amyloid Growth** | **3.60** | **0.89** | **<.0001** | **1.86** | **5.35** |
| Logical Memory | Baseline mean for Amyloid No Change class | 12.70 | 0.93 | <.0001 | 10.87 | 14.52 |
|  | Baseline mean for Amyloid Reduction class | 9.41 | 1.30 | <.0001 | 6.85 | 11.96 |
|  | Baseline mean for Amyloid Growth class | 9.40 | 0.77 | <.0001 | 7.88 | 10.92 |
|  | Annual rate of change for Amyloid No Change class | 0.63 | 0.14 | <.0001 | 0.35 | 0.91 |
|  | Annual rate of change for Amyloid Reduction class | 0.04 | 0.19 | 0.8253 | -0.33 | 0.42 |
|  | Annual rate of change for Amyloid Growth class | -0.04 | 0.12 | 0.7548 | -0.27 | 0.20 |
|  | **Amyloid Reduction vs Amyloid Growth** | **0.08** | **0.23** | **0.7236** | **-0.36** | **0.52** |
|  | **Amyloid No Change vs Amyloid Reduction** | **0.59** | **0.24** | **0.0134** | **0.12** | **1.05** |
|  | **Amyloid No Change vs Amyloid Growth** | **0.67** | **0.19** | **0.0003** | **0.30** | **1.03** |
| International Shopping  List Test | Baseline mean for Amyloid No Change class | 7.94 | 0.57 | <.0001 | 6.82 | 9.05 |
|  | Baseline mean for Amyloid Reduction class | 5.47 | 0.79 | <.0001 | 3.91 | 7.02 |
|  | Baseline mean for Amyloid Growth class | 5.32 | 0.47 | <.0001 | 4.40 | 6.25 |
|  | Annual rate of change for Amyloid No Change class | -0.13 | 0.08 | 0.1146 | -0.29 | 0.03 |
|  | Annual rate of change for Amyloid Reduction class | -0.21 | 0.11 | 0.0571 | -0.42 | 0.01 |
|  | Annual rate of change for Amyloid Growth class | -0.48 | 0.07 | <.0001 | -0.62 | -0.35 |
|  | **Amyloid Reduction vs Amyloid Growth** | **0.27** | **0.13** | **0.035** | **0.02** | **0.53** |
|  | **Amyloid No Change vs Amyloid Reduction** | **0.08** | **0.14** | **0.5522** | **-0.19** | **0.35** |
|  | **Amyloid No Change vs Amyloid Growth** | **0.35** | **0.11** | **0.0009** | **0.15** | **0.56** |
| Functional Assessment  Scale | Baseline mean for Amyloid No Change class | 1.93 | 0.88 | 0.0287 | 0.20 | 3.65 |
|  | Baseline mean for Amyloid Reduction class | 3.84 | 1.25 | 0.0023 | 1.38 | 6.30 |
|  | Baseline mean for Amyloid Growth class | 4.11 | 0.74 | <.0001 | 2.66 | 5.56 |
|  | Annual rate of change for Amyloid No Change class | 0.70 | 0.36 | 0.0563 | -0.02 | 1.41 |
|  | Annual rate of change for Amyloid Reduction class | 1.51 | 0.51 | 0.0031 | 0.51 | 2.51 |
|  | Annual rate of change for Amyloid Growth class | 2.53 | 0.31 | <.0001 | 1.92 | 3.14 |
|  | **Amyloid Reduction vs Amyloid Growth** | **-1.01** | **0.60** | **0.0891** | **-2.18** | **0.16** |
|  | **Amyloid No Change vs Amyloid Reduction** | **-0.82** | **0.62** | **0.1913** | **-2.05** | **0.41** |
|  | **Amyloid No Change vs Amyloid Growth** | **-1.83** | **0.48** | **0.0001** | **-2.77** | **-0.89** |


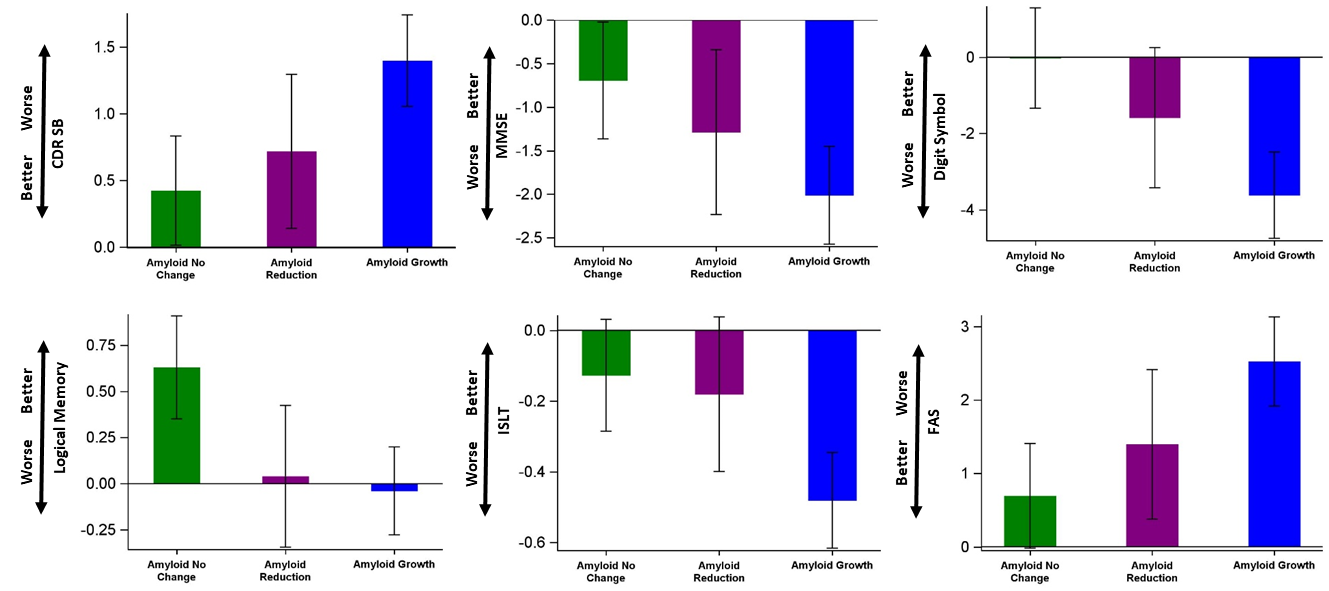


**Supplemental Figure** **2**: Estimated annual rate of change and 95% confidence interval (CI) by latent classes. 𝙸 bars (i.e., 95% CI) covering 0 indicate a non-significant rate of change at a two-sided type I error of 0.05. CDR SB, Clinical Dementia Rating Sum of Boxes; MMSE, Mini-Mental State Examination; Digit Symbol, Digit Symbol Substitution Test; Logical Memory, Logical Memory Delayed Recall Test; ISLT, International Shopping List Test-Delayed Recall; FAS, Functional Assessment Scale. The single placebo participant in the amyloid-reduction class was excluded.


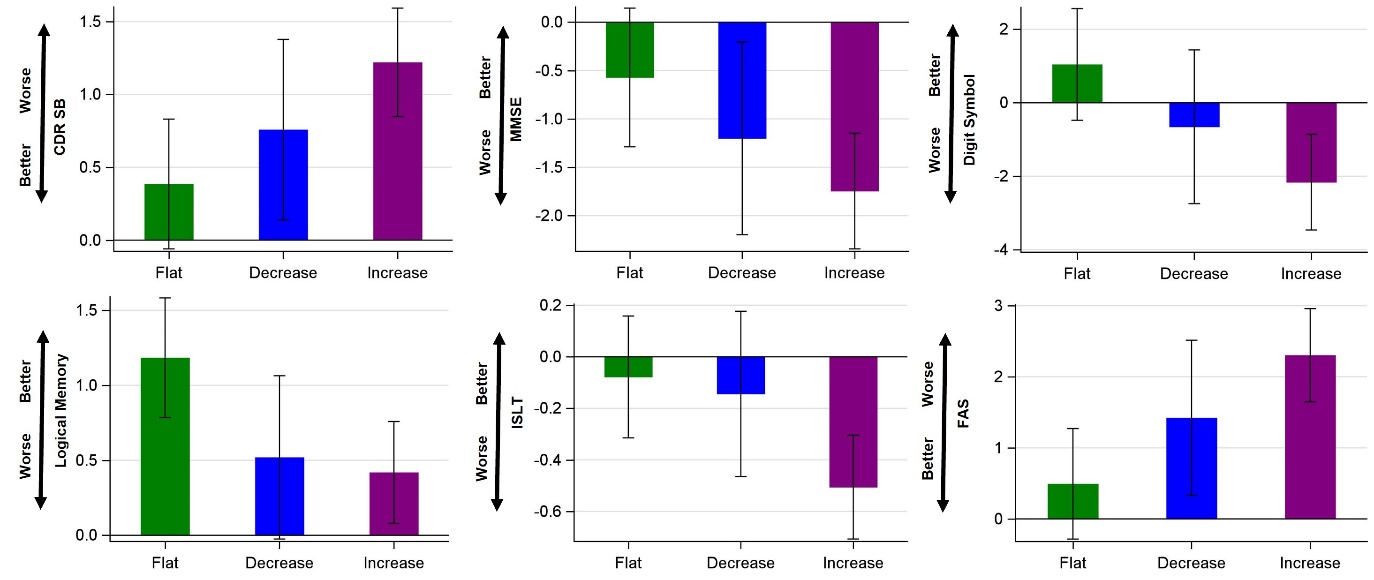


**Supplemental Figure** **3**: Estimated annual rate of change and 95% confidence interval (CI) by latent classes **using only the first 24 months of data**. 𝙸 bars (i.e., 95% CI) covering 0 indicate a non-significant rate of change at a two-sided type I error of 0.05. The amyloid-reduction class exhibited a reduction in the annual decline rate compared to the amyloid-growth class across multiple measures: CDR-SB decline was reduced by 37.8%, MMSE by 31.2%, digit symbol by 69.8%, ISLT by 71.5%, and FAS by 38.2%. CDR SB, Clinical Dementia Rating Sum of Boxes; MMSE, Mini-Mental State Examination; Digit Symbol, Digit Symbol Substitution Test; Logical Memory, Logical Memory Delayed Recall Test; ISLT, International Shopping List Test-Delayed Recall; FAS, Functional Assessment Scale.


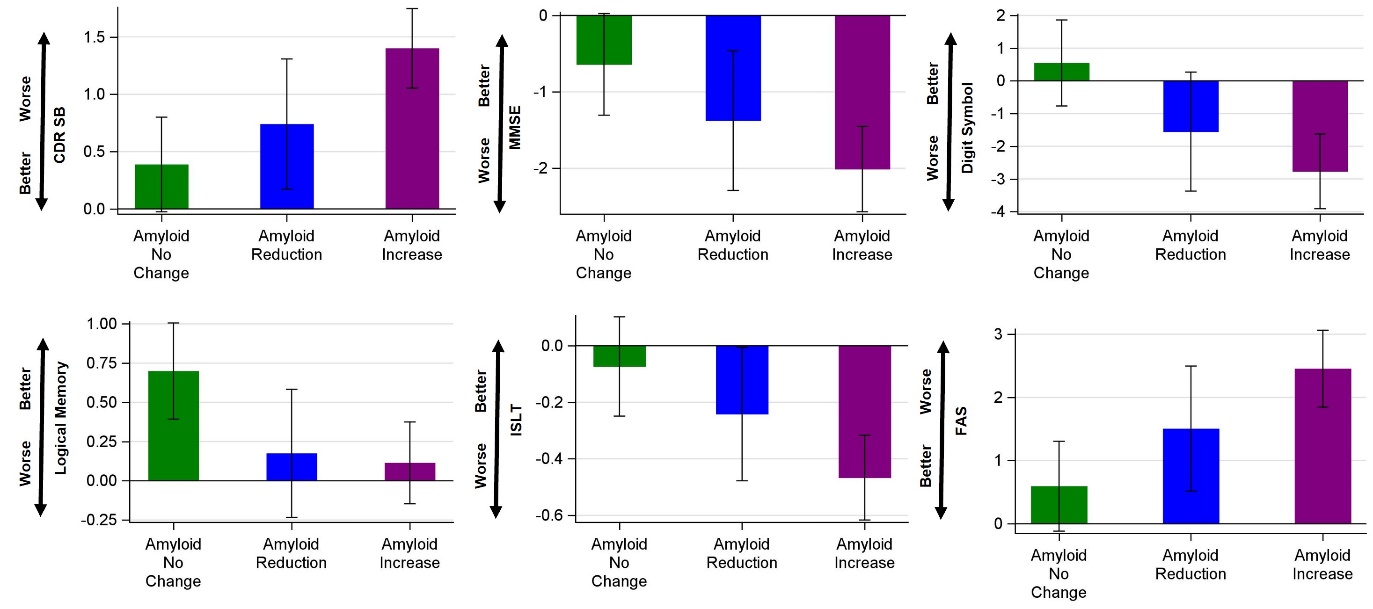


**Supplemental Figure** **4**: Estimated annual rate of change and 95% confidence interval (CI) by latent classes **with additional baseline covariates EYO and CDR global score**. 𝙸 bars (i.e., 95% CI) covering 0 indicate a non-significant rate of change at a two-sided type I error of 0.05. The amyloid-reduction class exhibited a reduction in the annual decline rate compared to the amyloid-growth class across multiple measures: CDR-SB decline was reduced by 47.2%, MMSE by 31.6%, digit symbol by 43.9%, ISLT by 48.2%, and FAS by 38.7%. CDR SB, Clinical Dementia Rating Sum of Boxes; MMSE, Mini-Mental State Examination; Digit Symbol, Digit Symbol Substitution Test; Logical Memory, Logical Memory Delayed Recall Test; ISLT, International Shopping List Test-Delayed Recall; FAS, Functional Assessment Scale.
