# Supplementary material for: Examining amyloid reduction as a surrogate endpoint through latent class analysis using clinical trial data for dominantly inherited Alzheimer's disease
Source: Alzheimers Dement. 2024 Feb 23;20(4):2698–706. doi: 10.1002/alz.13735 (PMC11032558; doi:10.1002/alz.13735)
Supplement: Supplementary file 3 — Supporting Information [file ALZ-20-2698-s002.docx]

**Appendix- Collaborators**

**DIAN-TU Study Team**

The DIAN-TU acknowledges the many individuals who have contributed to the DIAN-TU Trials including funding partners, leadership team, core leaders, project arm leaders, study sites and institutional study partners listed in the following pages.

**DIAN-TU Leadership Team**

Randall Bateman, MD, Director and Principal Investigator

Eric McDade, DO, Associate Director

David Clifford, MD, Associate Director and Medical Director

Stephen Salloway, MD, Project Arm Leader, Gantenerumab

Martin Farlow, MD, Project Arm Leader, Solanezumab

Lon Schneider, MD, Project Arm Leader

**DIAN-TU Core Leaders**

Randall Bateman, MD, Administrative Core Leader, Washington University School of Medicine

Anne Fagan, PhD, Biomarker Core Leader, Washington University School of Medicine

Chengjie Xiong, PhD, Biostatistics Core Leader, Washington University School of Medicine

Guoqiao Wang, PhD, Biostatistics Co-Core Leader, Washington University School of Medicine

Jason Hassenstab, PhD, Cognition Core Leader, Washington University School of Medicine

Alison Goate, DPhil, Genetics Core Leader, Mt. Sinai School of Medicine

Carlos Cruchaga, PhD, Genetics Core Leader, Washington University School of Medicine

Tammie Benzinger, MD, Imaging Core Leader, Washington University School of Medicine

Rick Perrin, MD, Neuropathology Core Leader, Washington University School of Medicine

Jorge Llibre-Guerra, MD, MSc, Post-Doctoral Associate

Cliff Jack, MD, MRI, Mayo Clinic

Robert Koeppe, MD, PET Imaging, University of Michigan

Nigel Cairns, MD (retired), Neuropathology Core Leader, Washington University School of Medicine

Peter Snyder, PhD, (former Cognition Core Leader), Brown University

**DIAN Expanded Registry (DIAN-EXR)**

Eric McDade - Director

Randall Bateman – Associate Director

Jorge Llibre-Guerra – Post-Doctoral Associate

Ellen Ziegemeier - Senior Clinical Research Coordinator

Jennifer Petranek - Clinical Coordinator I

Sarah Adams - Clinical Research Coordinator II

Susan Brandon - Clinical Research Coordinator IIDIAN-TU Staff

**DIAN-TU Faculty and Staff**

Amanda Fulbright, Grant Specialist, Administration Core

Ron Hawley, IT Audiovisual and Interactive web designer, Administration Core

Jacki Mallmann, Senior Grant Specialist, Administration Core

Karen McCann, Financial Accounting Assistant, Administration Core

Julie Murphy, Accounting/Purchasing Assistant, Administration Core

Anna Santacruz, Administrative Director, Administration Core

Jeanette Schillizzi, Research Administrator, Administration Core

Wendy Simpson, Financial Accounting Assistant, Administration Core

Shannon Sweeney, Finance Analyst, Administration Core

Kelley Coalier, Lead Scientist, Biomarker Core

Fatima Amtashar, QC Technician, Biomarker Core

Sushila Sathyan, Archivist, Biomarker Core

Jennifer Stauber, Research Specialist, Biomarker Core

Susan Mills, Director of Clinical Operations

Nicole Kelley, Associate Director of Clinical Operations

Stephanie Belyew, Clinical Trial Manager, Site, Vendor and GCP Management, Clinical Operations

Angela Fuqua, Clinical Trial Manager, Clinical Scale, Drug Supply and Vendor Management, Clinical

Operations

Inbal Meshulam, Clinical Trials Manager, Clinical Operations

Annette Stiebel, Clinical Trial Manager, Regulatory, Clinical Operations

Jeanine Portell, Clinical Trial Manager, MRI Sites, Clinical Operations

Bettina Bell, Clinical Trial Manager, Brain Donation, Clinical Operations

Caryll Bentley, Contract Project Manager, Clinical Operations

Sharon Cirello, Senior Contract Data Manager, Clinical Operations

Nithyanjali Devarapalli, Contract Specialist Clinical Data Manager, Clinical Operations

Arthur Gipson, Contract Specialist Clinical Data Manager, Clinical Operations

JaNeen Wisner, Contract Project Coordinator, Clinical Operations

Tayona Mayhew, Data Management Specialist, Clinical Operations

Zenobia Bridgewater, Clinical Research Coordinator, Clinical Operations

Dana Burgdorf, Research Nurse Coordinator II, Clinical Operations

Molly Fitzgerald, Clinical Research Study Assistant I, Clinical Operations

Erica Fowler, Clinical Research Coordinator I, Clinical Operations

Dottie Heller, Research Nurse Coordinator II, Clinical Operations

Miranda Jany, Clinical Research Coordinator I, Clinical Operations

Latoya Jones, Clinical Research Coordinator II, Clinical Operations

Michelle Jorke, Clinical Research Coordinator II, Clinical Operations

Paulette MacDougall, Research Nurse Coordinator II, Clinical Operations

Eugene Rubin, QC, Clinical Operations

Jessi Smith, Senior Clinical Research Coordinator, Clinical Operations

Mary Wolfsberger, Clinical Research Coordinator, Clinical Operations

Andy Aschenbrenner, Assistant Professor, Cognition Core

Jennifer Smith, Professional Rater III, Cognition Core

Marisol Tahan, Clinical Research Coordinator, Cognition Core

Theresa Butler, Research Lab Manager, Imaging Core

Lisa Cash, Senior Clinical Research Coordinator, Imaging Core

Jon Christensen, Staff Scientist, Imaging Core

Aylin Dince, Research Assistant, Imaging Core

Tony Durbin, Senior Clinical Research Coordinator, Imaging Core

Shaney Flores, Research Assistant, Imaging Core

Karl Friedrichsen, Research Assistant, Imaging Core

Brian Gordon, Co-Investigator, Imaging Core

Russ Hornbeck, Project Manager, Imaging Core

Nelly Joseph-Mathurin, Post-doc, Imaging Core

Sarah Keefe, Research Assistant, Imaging Core

Lakisha Lloyd, Research Coordinator, Imaging Core

Laura Marple, Research Technician II, Imaging Core

Austin McCullough, Graduate Research Assistant, Imaging Core

Stephanie Schultz, Pre-Doctoral Trainee, Imaging Core

Sally Schwarz, Co-Investigator, Imaging Core

Yi Su, Co-Investigator, Imaging Core

Andrei Vlassenko, Co-Investigator, Imaging Core

Qing Wang, Post-doc, Imaging Core

Jinbin Xu, Co-Investigator, Imaging Core

Erin Franklin, Research Coordinator, Neuropathology Core

**Eli Lilly and Company and Avid Radiopharmaceuticals, a wholly owned subsidiary of Eli Lilly**

**and Company**

Eli Lilly and Company Avid Radiopharmaceuticals

John Sims, MD, Eli Lilly and Company. Michael Devous

Karen Holdridge, MPH, Eli Lilly and Company. Erica Elephant

Cheryl Brown, BS, Eli Lilly and Company. Laura Harper

Roy Yaari, MD, Eli Lilly and Company Marybeth Howlett

Isabella Velona, Clinical Trial Project Manager Mark Mintun

Scott Andersen, Biostatistician Michael Pontecorvo

Michele Mancini, GPS

Brian Willis, PK/PD Project Leader

Jillian Venci Fuhs, Global Regulatory Affairs

Julie Bush, Product Delivery

Shamrock Garrett, Sr. CTMA

Traci Peddie, Data Sciences & Solutions

Natalie Vantwoud, Data Management

Barbara Lightfoot-Owens, Medical Writer

John Brad-Holmes, Central Lab

**Former Team Members**

Phyllis Ferrell Barkman, Russ Barton, Lauren Brunke, Robert Dean, Deanilee Deckard, Ann Catherine

Downing, Ganapathy Goppalrathnam, David Henley, Janice Hitchcock, Sonia Nijampatnam,Tracie

Peddie, Melissa Pugh, Tami Jo Rayle, Shiloh Scott, James Senetar, Gopalan Sethuraman, Eric

Siemers, Brian Steuerwald, Connie Tong, Jim Vandergriff

**F. Hoffman-LaRoche Team**

Monika Baudler, LifeCycle Leader

Rachelle Doody, Global Head of Neurodegneration

Paul Delmar, Principal Statistical Scientist

Carsten Hofmann, Clinical Pharmacologist

Michaela Jahn, Global Biometrics Team Leader

Geoff Kerchner, Global Development Leader

Gregory Klein, Biomarker Experimental Medicine Leader

Smiljana Ristic, Associate Group Medical Director

Alison Searle, Operations Program Leader

Marco Sonderegger, Technical Development Leader

Roz Sutton, EU Regulatory Partner

Janette Turner, US Regulatory Partner

Jaku Wojtowicz, Safety Science Director

Susan Yule, Global Regulatory Leader

**Former Team Members**

Elizabeth Ashford, Operations Program Leader; Bogdon Balas, Safety Science Leader; Estelle Vester-

Blokland, LifeCycle Leader; Stephanie Capo-Chichi, EU Regulatory Partner; David Agnew, Global

Study Manager; Ernest Dorflinger, Translational Medicine Leader; Efe Egharevba, Global Study

Manager; Christelle Laroche, Technical Development Leader; Isabelle Bauer Dauphin, Technical

Development Leader; Rob Lasser, Global Development Leader; Ferenc Martenyi, Global Development

Leader; Glenn Morrison, Global Development Leader; Tania Nikolcheva, Biomarker Experimental

Medicine Leader; Michael Rabbia, Statistical Scientist; Juha Savola, Project Leader; Janice Smith,

Clinical Science Leader; Dietmar Volz, Statistical Scientist

**DIAN-TU DSMB Members**

Gary Cutter, PhD, DSMB Chairperson, University of Alabama at Birmingham

Steve Greenberg, MD, PhD, Massachusetts General Hospital, Boston

Scott Kim, MD, PhD, National Institutes of Health, Bethesda

David Knopman, MD, Mayo Clinic, Rochester

Willis Maddrey, MD (retired), UT Southwestern, Dallas

Kristine Yaffe, MD, University of California, San Francisco

Karl Kieburtz, MD, PhD, (DSMB Chairperson, retired) University of Rochester, NY

Allan Levey, MD, PhD (retired), Emory University, Atlanta

**DIAN-TU Therapy Evaluation Committee**

Randall Bateman, MD, Chair, Washington University School of Medicine, St. Louis

Eric McDade, DO, Co-Chair, Washington University School of Medicine, St. Louis

Paul Aisen, MD, Alzheimer's Therapeutic Research Institute, USC

Jasmeer Chhatwal, MD, PhD, MMSc, Massachusetts General Hospital, Harvard Medical School

David Clifford, MD, Washington University School of Medicine, St. Louis

David Cribbs, MD, UC Irvine, CA

Nick Fox, MD, FRCP, FMedSci, Dementia Research Centre

Serge Gauthier, Serge Gauthier, CM, MD, FRCPC, Director, AD & Related Disorders Unit

McGill Centre for Studies in Aging

David Holtzman, MD, Washington University School of Medicine

Matthias Jucker, PhD, Hertie Institute for Clinical Brain Research, DZNE, Germany

Jeff Kelly, MD, Scripps University, California

Virginia Lee, PhD, University of Pennsylvania, Perelman School of Medicine

Simon Mead, FRCP, PhD, Institute of Prion Diseases, London

Cath Mummery, PhD, FRCP, Dementia Research Centre, London

Erik Musiek, MD, PhD, Washington University School of Medicine

Erik Roberson, MD, PhD, University of Alabama

Mathias Staufenbiel, PhD, Hertie Institute for Clinical Brain Research, DZNE, Tubingen, Germany

Robert Vassar, PhD, Northwestern University, IL

**Former TEC Members**

Bart DeStrooper, PhD; William Klunk, MD, PhD; Cynthia Lemere, MD; John C. Morris, MD

**DIAN Clinical Trials Committee (CTC) Members**

Randall Bateman, Washington University in St. Louis School of Medicine

John Morris, Washington University in St. Louis School of Medicine

Chengjie Xiong, Washington University in St. Louis School of Medicine

Denise Heinrichs, DIAN Family Representative

John Ringman University of California, Los Angeles

Laurie Ryan, Division of Science, National Institute on Aging

Neil Buckholtz, National Institute on Aging

Reisa Sperling, Director, Center for Alzheimer Research and Treatment

Stephen Salloway, Butler Hospital

Paul Aisen, University of California, San Diego

Anna Santacruz, Washington University in St. Louis School of Medicine

Gabrielle Strobel, Alzheimer Research Forum

Bill Klunk, University of Pittsburgh

William Thies, Alzheimer's Association

Anne Fagan, Washington University in St. Louis School of Medicine

Mark Mintun, Washington University in St. Louis School of Medicine

Natalie Ryan, University College London

Virginia Buckles, Washington University in St. Louis School of Medicine

David Hawver, Food and Drug Administration

Martin Farlow, Indiana University

Maritza Ciliberto, DIAN Family Representative

Ralph Martins, Edith Cowan University

Jennifer Williamson, Columbia University

**Study Sites**

**Australia**: Neuroscience Research Australia - W Brooks, MJ Fulham, J Bechara, D Foxe; Australian

Alzheimer’s Research Foundation – R Clarnette, N Reynders, P Mather; University of Melbourne – C

Masters, C Rowe, B Clinch, D Baxendale

**Canada**: McGill University – S Gauthier, P Rosa-Neto, C Mayhew, L Robb; University of British

Columbia – R Hsiung, D Worsley, M Assaly, E Nicklin; Sunnybrook Research Institute – M Masellis,

K Sharp, S Hetherington

**France:** Hopital Charles Nicolle – D Wallon, D Hannequin, A Morin, A Zarea, E Gerardin, P Bohn,

M Chastan, P Vera, M Colnot, N Donnadieu, M Quillard-Muraine, C Bergot, S Jourdain; Groupe

Hospitalier Pitie-Salpetriere – B Dubois, M Habert, N Younsi; Hopital Pierre Wertheimer – M

Formaglio, D Lebars, N El Kfif, A Jullien; Hopital Purpan – J Pariente, P Payoux, C Thalamas, A

Driff, E Pomies, P Gauteul; Hôpital Roger Salengro – F Pasquier, A Rollin-Sillaire, F Semah, L

Breuilh, M Laforce; Orsay Imaging – M Bottlaender

**Spain:** Hospital Clinic i Provincial de Barcelona – R Sanchez-Valle, M Balasa, A Lladó, B Bosch, N

Bargalló, I Banzo, A Perisinotti

**United Kingdom**: University College London Hospital – C Mummery, I Kayani, J Douglas, M Grilo

**United States:** Washington University School of Medicine – BJ Snider, T Benzinger, W Sigurdson, T

Donahue, P Kelly; Emory University – J Lah, C Meltzer, G Schwartz, P Vaughn, L Piendel;

University of Pittsburgh – S Berman, J Mountz, L Macedonia, S Ikonomovic, S Goldberg, E Weamer,

J Ruskiewiecz, S Hegedus, L Tarr, T Potter, G Valetti; University of Alabama at Birmingham – E

Roberson, D Geldmacher, M Love, A Watkins, L Ashley; Indiana University – J Brosch, A Kohn, N

McClaskey, J Buck, J Fletcher; Butler Hospital – G Surti, R Noto, C Bodge, W Menard; University of

Puerto Rico – I Jimenez Valazquez, J Diaz, K Aleman; Yale University – C van Dyck, M Chen, N

Diepenbrock, A Mecca, S Good; University of California San Diego – D Galasko, C Hoh, D Szpak, S

Peackock; University of Washington – S Jayadev, D Lewis, Y Tutterow

**DIAN-TU Collaborators and Advisers**

John Morris, MD, Senior Advisor, Washington University School of Medicine, St. Louis, MO

David Holtzman, MD, Senior Advisor, Washington University School of Medicine, St. Louis, MO

Laura Swisher, MS, Deputy Director, Washington University School of Medicine, St. Louis, MO

Alisha Daniels, MD, MHA, Executive Director, DIAN, Washington University School of Medicine,

St. Louis, MO

Janice Hitchcock, PhD, Hitchcock Regulatory Consulting Inc.

Thomas Bird, MD, University of Washington, Seattle

Dennis Dickinson, MD, Mayo Clinic, Jacksonville, FL

M. Marsel Mesulam, MD, Cognitive Neurology and Alzheimer’s Disease Center, Northwestern

University, Chicago, IL

Cornelia Kamp, MBA, University of Rochester, New York

Ron Thomas, PhD, ADCS, University of California, San Diego

Paul Aisen, MD, ADCS, University of Southern California, Los Angeles

**DIAN Observational Study Site Investigators**

James Noble, MD, Columbia University, New York

Martin Farlow, MD, Indiana University, Indianapolis, IN

Jasmeer Chhatwal, MD, PhD, Brigham and Women’s Hospital-Massachusetts GH, Charlestown, MA

Stephen Salloway, MD, Butler Hospital, Warren Alpert School of Medicine, Brown University

Sarah Berman, MD, PhD, University of Pittsburgh, PA

Gregg Day, MD, Mayo Clinic Jacksonville, FL

Hiroyuki Shimada, MD, PhD, Osaka City University, Japan

Takeshi Ikeuchi, MD, PhD, Brain Research Institute, Nigata, Japan

Kazushi Suzuki, MD, PhD, The University of Tokyo, Japan

Peter Schofield, PhD, DSc, Neuroscience Research Australia, Sydney

Ralph Martins, BSc, PhD, Edith Cowan University, Nedlands, Western Australia

Nick Fox, MD, FRCP, FMedSci, Dementia Research Centre, University College London, United

Kingdom

Johannes Levin, MD, PhD, German Center for Neurodegenerative Diseases (DZNE), Munich, Germany

Mathias Jucker, PhD, German Center for Neurodegenerative Diseases (DZNE), Tubingen, Germany

Raquel Sanchez Valle, MD, Hospital Clinic i Provincial de Barcelona, Spain

Patricio Chrem, MD, Fundación para la Lucha contra las Enfermedades Neurológicas de la Infancia

(FLENI), Buenos Aires, Argentina

**DIAN EXR Referring Clinicians, Researchers and Partner Sites**

Neelum T. Aggarwal, MD, Rush University Medical Center, Chicago IL

Tom Ala, Center for Alzheimer’s Disease and Related Disorders, Southern Illinois University School

of Medicine

Thomas Bird, University of Washington, Seattle

Sandra E. Black, Sunnybrook Health Sciences Centre, University of Toronto, Canada

William J. Burke, MD, Banner Alzheimer’s Institute

Cynthia M. Carlsson, MD, MS, University of Wisconsin School of Medicine and Public Health

Andrew Frank M.D. B.Sc.H. F.R.C.P.(C), Bruyere Continuing Care, Ottawa, Ontario, Canada

James E. Galvin, MD, MPH, Charles E. Schmidt College of Medicine, Florida Atlantic University

Alvin C Holm, MD, Bethesda Hospital, St. Paul, MN

John S.K. Kauwe, Brigham Young University

David Knopman MD, Mayo Clinic, Rochester MN

Sarah Kremen, MD, University of California, Los Angeles

Alan J. Lerner, University Hospitals Cleveland Medical Center

Barry S. Oken, MD, PhD, Oregon Health & Science University

Hamid R. Okhravi, Eastern Virginia Medical School

Ronald C. Petersen, Mayo Clinic, Rochester, MN

Aimee L. Pierce, MD, University of California Irvine

Marsha J. Polk, MED, University of Texas Health Science Center at San Antonio

John M. Ringman, MD, MS, University Southern California

Peter St. George Hyslop, MD, FRS, FRSC, FRCPC, University of Toronto

Sanjeev N. Vaishnavi, MD, PhD, University of Pennsylvania

Sandra Weintraub, Northwestern University Feinberg School of Medicine, IL
